# Nursing mothers’ social representations regarding sexualities in the breastfeeding context: a correspondence factor analysis*

**DOI:** 10.1590/1980-220X-REEUSP-2024-0162en

**Published:** 2024-12-02

**Authors:** Ana Beatriz Azevedo Queiroz, Edilene Macedo Cordeiro Figueiredo, Ana Luiza de Oliveira Carvalho, Juliana da Fonsêca Bezerra, Elen Petean Parmejiani, Maria Sagrario Gómez Cantarino, Maria Ludmila Kawane de Sousa Soares, Aline Furtado da Rosa

**Affiliations:** 1 Universidade Federal do Rio de Janeiro, Escola de Enfermagem Anna Nery, Rio de Janeiro, RJ, Brazil.; 2 Fundação Universidade Federal De Rondônia, Departamento De Enfermagem, Porto Velho, Ro, Brazil.; 3 Universidad de Castilla-La Mancha, Facultad de Fisioterapia y Enfermería, Campus de Toledo, Spain.

**Keywords:** Sexuality, Breastfeeding, Reproductive Health Services, Social Psychology, Social Representation, Sexualidade, Aleitamento Materno, Serviços de Saúde Reprodutiva, Psicologia Social, Representação Social, Sexualidad, Lactancia Materna, Servicios de Salud Reproductiva, Psicología Social, Representación Social

## Abstract

**Objective:**

To analyze nursing mothers’ social representations of sexuality in the breastfeeding context and their repercussions in sexual and reproductive health.

**Method:**

An exploratory study grounded on the Theory of Social Representations and conducted in a Human Milk Bank from northern Brazil with 110 nursing mothers, following the Free Word Association Technique. Data were analyzed by means of Correspondence Factor Analysis in the software Tri- Deux-Mots 5.1.

**Results:**

Sexual intercourse was the significant expression of sexualities, reflected in the representation of this phenomenon in breastfeeding, anchored in cultural gender relations; meanwhile, the search for sexual desire and pleasure connected to the ideal of romantic love arises. The bodily and daily changes inherent to this period take on an unfavorable connotation and become naturalized over time, negatively influencing nursing mothers’ sexual and reproductive health.

**Conclusion:**

Understanding sexuality/sexualities in the breastfeeding context can support Nursing and Health care models in line with the social experiences and thoughts of the different groups of nursing mothers.

## RESUMO

**Objetivo:**

Analisar as representações sociais de nutrizes sobre sexualidade no contexto da amamentação e suas repercussões na saúde sexual e reprodutiva.

**Método:**

Estudo exploratório fundamentado na Teoria das Representações Sociais, realizado em um Banco de Leite Humano da região Norte do Brasil com 110 nutrizes, seguindo a Técnica de Associação Livre de Palavras. Os dados foram analisados por meio da Análise Fatorial de Correspondência no software Tri-Deux-Mots 5.1.

**Resultados:**

A relação sexual foi a expressão significativa das sexualidades, refletida na representação desse fenômeno na amamentação, ancorada nas relações culturais de gênero; ao mesmo tempo, surge a busca pelo desejo e prazer sexual conectado ao ideal do amor romântico. As mudanças corporais e cotidianas inerentes a esse período assumem uma conotação desfavorável e foram naturalizadas ao longo do tempo, influenciando negativamente a saúde sexual e reprodutiva das nutrizes.

**Conclusão:**

Compreender a sexualidade/sexualidades no contexto da amamentação pode embasar modelos de assistência de Enfermagem e Saúde alinhados às experiências e pensamentos sociais dos diferentes grupos de mães que amamentam.

## RESUMEN

**Objetivo:**

Analizar las representaciones sociales de la sexualidad de las madres lactantes en el contexto de la lactancia materna y sus repercusiones en la salud sexual y reproductiva.

**Método:**

Estudio exploratorio fundamentado en la Teoría de las Representaciones Sociales y realizado en un Banco de Leche Humana del norte de Brasil con 110 madres lactantes, siguiendo la Técnica de Asociación Libre de Palabras. Los datos fueron analizados mediante Análisis Factorial de Correspondencia en el software Tri- Deux-Mots 5.1.

**Resultados:**

La relación sexual fue la expresión significativa de las sexualidades, reflejada en la representación de este fenómeno en la lactancia materna, anclada en las relaciones culturales de género; entretanto, surge la búsqueda del deseo y placer sexual ligado al ideal del amor romántico. Los câmbios corporales y cotidianos propios de este periodo adquieren una connotación desfavorable y se naturalizan con el tiempo, influyendo negativamente en la salud sexual y reproductiva de las madres lactantes.

**Conclusión:**

Comprender la sexualidad/las sexualidades en el contexto de la lactancia materna puede apoyar modelos de Enfermería y Atención de Salud acordes con las experiencias y pensamientos Sociales de los diferentes grupos de madres lactantes.

## INTRODUCTION

Sexuality is the result of a vital impulse present in the life of every individual in a subjective way; it is considered that its expression is manifested in different ways in different social contexts^(1)^. Therefore, the way in which sexuality will emerge depends on the sociocultural condition in a given historical period, on the established gender, and on social relations, as it represents the roles assumed in society that govern human thoughts and behaviors^(2)^.

It does not regard only human sexuality, which is multifactorial, nor is only viewed from a biological point of view, but also includes psychological elements, social, political and cultural construction, in addition to legal, religious and spiritual influence. In this sense, aspects such as sex, identities, gender roles, sexual orientation, eroticism, intimidated pleasure, and reproduction are related to the theme, and cannot be excluded from this interaction^(3)^.

By definition, breastfeeding is the condition of feeding an infant directly from the mother’s breast, expressing a moment of emotional interaction between them and which, for nursing mothers, represents an experience that interferes with many dimensions of their lives, including their sexuality^(4)^. Breastfeeding is still strongly propagated in our society as a maternal, sacred and asexual practice, based on the nutritional, biological, and functional aspects aimed at the child’s physical well-being^(5)^.

Still with the inheritance of a social construction based on patriarchy, the woman who breastfeeds is given the duty of donation, considering that feeding one’s own child is divine and the result of this is the sacralization of breastfeeding. From a health point of view, there are still strong marks of a biomedical paradigm, where the focus of breastfeeding is eminently nutritional and therefore other aspects are not included in this discussion, such as whether the woman actually wants to breastfeed and sexuality^(6)^.

Consequently, the construction of sexuality in the breastfeeding experience is influenced by cultural, biological, social, and historical factors, in addition to each nursing mother’s world view. The interface between these two phenomena can be understood based on the representation of the breasts, which, in the Western culture, have two major elaborations: the nurturing breast and the erotic breast, an important expression of female sexuality^(6)^. This symbolic construction of the breasts contributes to nursing mothers feeling divided between the role of asexual mothers and sexual women, bringing about the symbolism of the pure and the profane^(7)^.

In fact, nursing mothers face a dichotomy between the mother and woman roles, which allows experiencing different senses that guide behaviors and actions regarding sexualities. National and international studies prove that nursing mothers end up looking for alternatives to experience their return to active sexual life, such as the option for the horizontalized division of the female body, with the upper part belonging to the infant and the lower part, represented by the sexual organ, to the partner^(5,6,7,8)^.

This duality can result in difficulties for nursing mothers in the process of reconciling their female sexuality and continuity of the breastfeeding process^(9)^. With this, nursing mothers’ sexuality in the breastfeeding period is immersed in cultural contexts and is socially elaborated in a network of meanings inherent to the social group in which women are inserted. Thus, from a perspective of professional qualification and of subsidies for comprehensive health care strategies, it becomes necessary to understand the context that permeates sexuality in the breastfeeding period, as nursing mothers’ attitudes and behaviors in the face of this phenomenon involve subjectivities, diverse knowledge that circulate in the social environment, information sources, values and beliefs.

In seeking to understand this phenomenon from a psychosociological perspective and based on the premise that social representations investigate how people interpret reality and elaborate explanations for social objects^(2)^, the objective of this study is to analyze the social representations (SRs) of sexuality in the breastfeeding context for nursing mothers and their repercussions on sexual and reproductive health.

When considering human sexualities, especially the sexualities of breastfeeding women, it is necessary to understand that these are transmissions of cultural and socially constructed meanings and that they are integrated into a network of meanings of the social group^(6)^, resulting in the elaboration of social representations for women.

Therefore, let us understand that the sexualities of nursing women in the context of breastfeeding are a topic that has cultural and social relevance, as it produces different approaches from the universe of common sense, being based on meanings and senses that allow evoking social representations about it.

## METHOD

### Type of Study

This is an exploratory study guided by the Theory of Social Representations (SRTs), according to Moscovici^(2)^. This framework was chosen because it allows for a deeper understanding of the universe of meanings inherent to human actions and relationships, which permitted understanding nursing mothers’ social thinking about sexuality in the breastfeeding context. SRs metabolize the novelties that the world presents to us, returning to it in the form of understanding, but also as judgments, definitions, and classifications^(2)^. Their functions are to explain reality, define group identity, guide practices, and justify stances taken^(10)^.

### Local

The study was conducted at a Human Milk Bank (HMB) located in northern Brazil, in the municipality of Porto Velho, Rondônia. This institution is a reference in promoting and encouraging breastfeeding in the state, providing care and counseling in this area by duly qualified professionals.

### Participants

The inclusion criteria corresponded to nursing mothers aged over 18 years old, from the 45^th^ postpartum day, living with their partners, and registered in the HMB. No exclusion criteria were defined.

### Data Sources

The nursing mothers were invited to participate in the research during their consultations at the HMB and, after presenting the study objectives and stages, those who agreed to participate were taken to an interview in a private environment within the institution, without any risk of exposure or interruptions.

### Data Collection, Organization and Analysis

Data collection took place from June 2020 to June 2021 by means of the Free Word Association Technique (FWAT) and using an instrument with the purpose of obtaining the socioeconomic, demographic, and sexual and reproductive health profiles,

FWAT is a projective technique that acts directly on the psychological structure of individuals by inducing stimuli, which can be verbal or non-verbal and whose responses seek to highlight the representations about the inducing object. This technique enables apprehending the evocation of ideas from a social group, revealing implicit or latent contents, and is widely used for research studies on SRs^(11)^.

For the survey in question, the two inducing stimuli, called “opinion variables” in the technique, were the following: sexuality, and sexuality in the breastfeeding period. These terms were chosen because they are easy to understand, for meeting the research objective, and for being close to the participants’ language. The participants wrote up to three words in an exclusive instrument for each inducing stimulus. The first guiding question from the FWAT was as follows: What comes to your mind when I talk about sexuality? Once all the answers to this first question had been given, the second question was asked: What comes to your mind when I talk about sexuality in the breastfeeding period?

Data were analyzed using the Tri-Deux-Mots 5.1 software to select the quali-quantitative data resulting from using FWAT^(12)^, as it allows for an interpretation based on Correspondence Factor Analysis (CFA), a multivariate descriptive statistics technique which highlights the affinities between certain rows and columns of a data matrix. It is based on the hypothesis of independence between the rows and columns of this same table^(13)^. It therefore allows for a graphic representation of the approximation, distance, confrontation, and attraction that are formed between the opinion variables, that is, the evoked words (lines) and the fixed variables (columns) that identify the subjects^(11)^.

The database to be processed in the software was initiated after data collection, and a coding was created for both the fixed variables used in the study, namely: nursing mother’s age (AGE), parity (PAR), and time as a nursing mother (TIM), and for the opinion variables: sexuality (SEX) and sexuality during breastfeeding (SEXBF), as shown in the Chart 1 below.

**Chart 1:** Coding of the fixed and opinion variables used to comprise de database prior to processing in the Tri-Deux-Mots software – Porto Velho, RO, Brazil, 2021.

FIXED VARIABLES		
NURSING MOTHER’S **AGE**	PARITY **PAR**	TIME AS A NURSING MOTHER **TIM**
1) 18–29 years old 2) 30–40 years old	1) PRIMIPAROUS 2) MULTIPAROUS	1) UP TO 6 MONTHS 2) 7 MONTHS – 1 YEAR 3) MORE THAN 1 YEAR
**OPINION VARIABLES**		
1. SEXUALITY **SEX**	2. SEXUALITY IN THE BREASTFEEDING PERIOD **SEXBF**	

**Source:** Research data, 2021.

All the information contained in the database was imported into the software, where the factor calculation stage (factor 1 and factor 2) was performed, referring to the contribution of the fixed and opinion variables in CFA.

The Tri-Deux-Mots indicates the evoked words of greater significance for the construction of Factors 1 and 2 from the factor plan, generating the Factor Graph, which shows the relationship of fixed variables with opinion variables and their contribution in the construction of these factors through the Contribution per Factor (CPF) number, allowing the identification of the most relevant variables for the formation of the factor plan^(13)^. As a criterion for evaluating the factor representation quality performed in Tri-Deux-Mots the sum of factors 1 and 2 (F1 + F2) is used and, when the sum of both factors explains at least 15% of the total variance, it is considered a good quality analysis. In this study, the sum of factors 1 and 2 explains 76% of the total variance in the answers, being considered an optimum margin analysis^(11)^.

In studies presenting the CFA for the apprehension of SRs, it is agreed that axis 1 (Factor 1) occupies the horizontal position of the graph with the evocations represented in red, whereas axis 2 (Factor 2) occupies the vertical position with the evocations in blue^(11)^. As a way to reduce data dispersion in the factor plan, it was decided that the analysis of the opinion variables having a CPF equal to or greater than 30 would be established.

After generating the graph, it was saved in *Paint* to make the necessary edits, such as evidencing the axis quadrants and highlighting the variables with the terms evoked. Finally, interpretation and analysis of the data obtained in CFA were based on the TSR.

### Ethical Issues

The study was approved by the Ethics Committee of the Anna Nery Nursing School (*Escola de Enfermagem Anna Nery*, EEAN) and the São Francisco de Assis Health Care Center belonging to *Universidade Federal do Rio de Janeiro*, Brazil, under opinion No. 83.035.088. All the participants signed the Free and Informed Consent Form (FICF), thus recording their acceptance. None of the nursing mothers invited refused to take part in the research.

## RESULTS

The participants were 110 nursing mothers assisted in the aforementioned HMB, through simple random sampling and in the universe of 220 nursing mothers treated in this setting during the collection period.

The nursing mothers’ age group varied between 18 and 40 years old, with 69% aged between 18 and 29. In relation to self-declared race/skin color and religiousness, there was a predominance of the black race (85%) and of Evangelicals (59%), and 71% were active in the labor market with family incomes between 1 and 3 minimum wages, considered as low income in the Brazilian context^(14)^.

When analyzing the sexual and reproductive health profile, it was identified that most of the participants were primiparous (52%), with unplanned pregnancies (61%), and having undergone C-sections (64%). In relation to their time as nursing mothers, 40% were so for less than six months, 30% from 6 to 12 months, and other 30% from 13 to 24 months. Regarding their sexual activities, 78% had resumed their sexual life, 65% said they had no desire or pleasure in sexual relations. As for the use of contraceptive methods, 47% did not use any, despite their non-intention to get pregnant at that moment.

The analysis of the material obtained by the FAWT showed that 492 words related to the inducing terms were evoked, with 176 different words, of which 26 contributed to organization of the factor plan and allowed approaching the consensual contents shared by the nursing mothers in the field of SRs on sexuality in the breastfeeding period.

**Figure 1:**
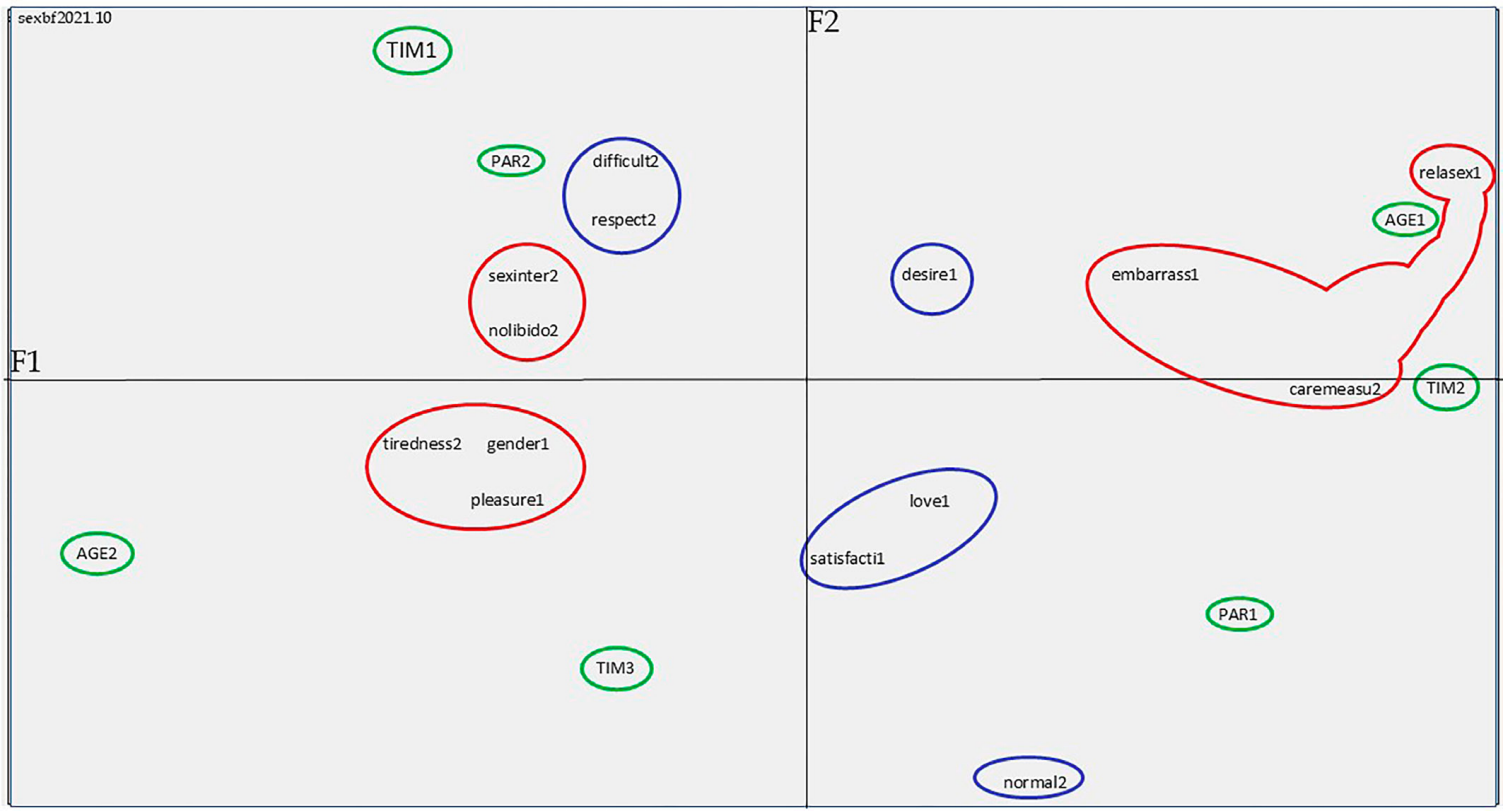
Correspondence Factor Plan of the Social Representations elaborated by the nursing mothers on sexualities, Porto Velho, Rondônia, Brazil, 2021. **KEY:** F1 axis: It is the axis that corresponds to the right (F1+) and left (F1–) sides of Figure 1. F2 axis: It is the axis that corresponds to the upper (F2+) and lower (F2–) sections of Figure 1.

**Source:** Tri-Deux-Mots report.

From the analysis of the factor plan corresponding to the SRs of the nursing mothers’ sexualities and considering the most significant evocations and their respective CPF values, it was possible to identify that, in relation to the *sexuality* inducing stimulus, the term that most contributed to constructing the factor was **sexual intercourse** (CPF = 200). In relation to the second inducing stimulus: *sexuality in the breastfeeding period*, the evocation with the highest contribution was **normal** (CPF = 353). However, these evocations presented differences in relation to the stimulus when cross-analyzed with the fixed variables, as can be seen in Figure 1 and in Chart 2.

**Chart 2:** Nursing mothers’ evocations based on the inducing stimuli and according to age, time as a nursing mother, and parity – Porto Velho, Rondônia, Brazil, 2021.

SEXUALITY				SEXUALITY IN THE BREASTFEEDING PERIOD			
**18 – 29 YEARS OLD (AGE1)**		**30 – 40 YEARS OLD (AGE2)**		**18 – 29 YEARS OLD (AGE1)**		**30 – 40 YEARS OLD (AGE2)**	
SEXUAL INTERCOURSE		GENDER PLEASURE		EMBARRASSMENT CARE MEASURES		TIREDNESS SEXUAL INTERCOURSE NO LIBIDO	
**≤6 MONTHS (TIM1)**	**7 MONTHS – 1 YEAR (TIM2)**		**>1 YEAR (TIM3)**	**≤6 MONTHS (TIM1)**	**7 MONTHS – 1 YEAR (TIM2)**		**>1 YEAR (TIM3)**
DESIRE	SEXUAL INTERCOURSE		LOVE SATISFACTION	RESPECT DIFFICULT	EMBARRASSMENT CARE MEASURES		NORMAL
**PRIMIPAROUS (PAR1)**		**MULTIPAROUS (PAR2)**		**PRIMIPAROUS (PAR1)**		**MULTIPAROUS (PAR2)**	
LOVE SATISFACTION		DESIRE		NORMAL		RESPECT DIFFICULT	

Figure 1 represents the factor plan resulting from the Tri-Deux-Mots analysis, consisting of the F1 (horizontal) and F2 (vertical) axes: it presents the evocations and the relationship with the fixed variables circled in green.

Chart 2 below presents the comparative data between the most relevant evocations that emerged from both inducing terms, analyzed based on the three fixed variables.

In the first stimulus, *sexuality*, on the horizontal axis (F1), the evocation that was most representative, and its CPF on the right side (F1+) was **sexual intercourse** (CPF = 200), which is related to younger nursing mothers aged between 18 and 29 years old (AGE1, CPF = 251) and with nursing mothers with between 7 months and 1 year as such (TIM2, CPF = 207). As for the left side (F1–), the most representative words in its construction according to CPF were **gender** (CPF = 73) and **pleasure** (CPF = 66), which are related to adult women aged between 30 and 40 years old (AGE2, CPF = 278).

In the vertical axis (F2), the most representative word evoked in the upper section (F2+) was **desire** (CPF = 33), which is related to nursing mothers with a maximum of six months as such (TIM1, CPF = 261) and to the multiparous participants (PAR2, CPF = 153). In the lower section of the vertical axis (F2–), the words that most contributed to the formation of this axis were as follows: **satisfaction** (CPF = 63) and **love** (CPF = 40) for nursing mothers with more than one year as such (TIM3, CPF = 237) and for the primiparous participants (PAR1, CPF = 163).

Regarding the second stimuli, *sexuality in the breastfeeding period*, the right side of the F1 axis, which refers to F1+, had the following words as the most representative ones: **care measures** (CPF = 181) and **embarrassment** (CPF = 60), which were related to the group of younger women (AGE1, CPF = 251) and to nursing mothers with 7 months to 1 year as such (TIM2, CPF = 207). The most frequently evoked words in the left side of the axis (F1-) were as follows: **tiredness** (CPF = 107), **sexual intercourse** (CPF = 45), and **no libido** (CPF = 52), which were associated with the group of women aged from 30 to 40 years old (AGE2, CPF = 278).

In the upper section of the F2 vertical axis (F2+), the words that most contributed to constructing this axis were the following: **difficult** (CPF = 150) and **respect** (CPF = 77), presenting an association with nursing mothers with up to six months as such and with the multiparous participants. Regarding the lower section of the F2 axis (F2–), the most representative word was **normal** (CPF = 353) and was related to nursing mothers with more than 1 year as such (TIM3, CPF = 150) and to the primiparous participants (PAR1, CPF = 60).

## DISCUSSION

For the nursing mothers, the construction of sexuality/sexualities emerged objectified in the reductionism of sexual intercourse, mainly in the context of the younger ones and nursing mothers of 7 months – 1 year, presenting a physical and embodied connotation, sustained on genitality and on the sexual organs. Referring to this aspect, a study conducted in the United States found that, even today, women have a tendency to delimiting sexuality to genital experiences. It asserts that this thought can be related to a male perspective of the meaning of sexuality, which establishes genital development as the final objective and, more importantly, of the maturation of human sexuality^(15)^. This reductionism can contribute to limiting nursing mothers’ satisfaction with their sexuality, given the physiological and endocrine issues that may reduce libido and the scope of pleasure to genital manipulation or penetration.

Regarding these endocrine and physiological issues, prolactin stands out, which has a lactogenic effect on milk, that is, with high levels of prolactin during the breastfeeding period, there is a reduction in gonadotropic hormones (LH and FSH) and this results in decreased libido and anovulatory periods. In addition to all the changes expected in the female body during breastfeeding, the new routine of caring for the newborn, care with household chores, absence of a support network, lack of guidance during prenatal care, can also interfere with female libido^(15)^.

However, the other segments of participants, adults, multiparous/primiparous mothers, nursing mothers up to 6 months and over a year, elaborate sexuality with senses of pleasure, desire and satisfaction. It is verified that sexual intercourse encompasses elaborations that do not merely limit it to the act itself, with the emergence of senses such as pleasure, desire, and satisfaction. This change in the connotation attributed to sexual intercourse can be anchored in a historical context of modification of the perspective about female pleasure that has been taking place over the last few years, resulting from women’s search for their desires and assuming a leading role in their social relations, which includes sexual practices to obtain pleasure^(16)^.

Desire and pleasure are important forms of expression of human sexualities, consisting of the elaboration of fantasies and desires for sexual satisfaction^(16)^. It is inferred that the SRs of sexuality/sexualities elaborated by these segments of nursing mothers present an affective dimension, when they refer to the existence of these feelings associated with sexuality/sexualities.

The evocation of the word “gender” arises as a symbolic construction of sexuality/sexualities for the adult nursing mothers, evidencing the cognitive dimension that comprises the SRs of this segment. This term brings about the representativeness of the association between gender-based relations and sexuality, which can be supported by the social recognition of inequalities between men and women^(17)^. This acknowledgment is due to women leaving the exclusive private domestic space, in addition to their access to education and the labor market bringing about ideals of female empowerment in the struggle for their sexual autonomy, in the sense of having freedom and living their sexuality in a pleasurable way^(16)^, which should be reinforced and encouraged in the sexual and reproductive health care scope.

In this research, the empirical data also indicate that the representation of sexuality/sexualities is anchored in romantic love. The dream of finding an ideal partner and of establishing relationships in a romantic context is something that, even today, women learn throughout female education^(18)^. In this axis, it can be identified that these women develop their sexuality(s) in the idealization of a love relationship, evidencing the symbolic and affective dimension of SRs.

A study on sexuality during breastfeeding uses the term sacralization of breastfeeding, that is, the female body is socially disallowed from enjoying feelings of intimacy and eroticism. Furthermore, the silence of women makes sexuality in breastfeeding invisible^(6)^.

Even facing the perspective of sexuality/sexualities interpreted as a sexual relationship with desire and pleasure, a number of research studies increasingly corroborate a comprehensive understanding of sexual satisfaction coexisting with affective-love satisfaction, mainly for women. They point out that breaking this paradigm of sexual-love-pleasure coexistence implies a change in the social roles attributed to the genders, which still constitutes a major challenge^(6,17)^.

This association of satisfaction and love was more intense among the nursing mothers with more than a year as such and among the primiparous women, showing that the individuals’ social place is essential in the production of SRs, as it reveals institutional, ideological, political and cultural conditions that portray the social mark of the representations^(2,10)^.

Faced with the explanation and definition of sexuality/sexualities, it is possible to understand the elaborations that form the SRs of sexuality/sexualities during the breastfeeding period, although with specific dimensions and meanings of the experience as nursing mothers for women. The evocations of embarrassment and care measures bring about the representation of a female body that is embarrassed or intimidated by the changes that occurred during pregnancy, delivery and, mainly, breastfeeding, as a body that is no longer attractive and seductive, and which may even be prohibited from having sex^(18)^. These representations are anchored in the aesthetic standard socially conditioned to the feminine, in which a woman’s body needs to be aligned with the ideals of beauty, so that she feels contemplated in the identity construction of being a woman^(19)^.

The bodily changes imposed by the pregnancy-puerperal cycle seem to come against this ideal of beauty, where a woman’s identity is oftentimes lost, becoming a foreigner in her own body^(20)^. In this perspective, this representation was strongly present in the context of younger nursing mothers and those with breastfeeding times between 7 months and 1 year, whose desire to return to a body within the beauty and youthfulness standards seems to be more surfaced.

A number of studies^(8,19,21)^ point out that these changes and the absence of body identity related to breastfeeding and pregnancy are considered factors that cause shame, discomfort, and implications for nursing women’s self-esteem. Therefore, when caring for nursing mothers, it is important to seek strategies to value their bodies in their current state and improve their self-esteem, as embarrassment with their bodies, in addition to dissatisfaction with their sex lives, can result in harms to breastfeeding.

The adult participants sought to explain the meaning of sexuality/sexualities during the breastfeeding period based on tiredness and sexual intercourse without libido, which allows inferring that sexuality/sexualities in this life phase are surrounded by exhaustion and lack of desire, suggesting the representation of a sexual life out of obligation, when it turns out that 65% report neither desire nor sexual pleasure.

Both historically and culturally, sex for women is understood as exempt from emotions and pleasure with the mere purpose of procreation, making female nature demand marriage, stipulating rules and norms for women, such as serving their partner’s pleasure^(17)^. In this context, contemporaneously and even if with less ideological force, women still carry with them this function to sexual life, being subjugated in their wills and desires. This representation is anchored in the cultural memory of gender inequality rooted in the patriarchal culture that values men and places them in a higher social condition, conferring them the right to imprint their wills and decisions in the family context, keeping women away from what they really want for themselves, even in the sexual sphere^(16)^.

A number of research studies on this phase of women’s life, carried out in Germany, the United States, France and also Brazil, corroborate the findings that evidence a reduction in sexual practices without pleasure resulting from decreased libido, especially for women who have other children, who work outside home and still accumulate household chores, due to changes in their daily lives^(8,15,21,22,23)^. These changes are proven by these studies as crisis and imbalance situations that affect all life aspects, including attitudes, as all behaviors are impregnated with meanings, enriching the context of what experienced reality is for each person^(2)^.

In this line of reasoning, it is possible to understand that tiredness emerged as one of the meanings attributed to sexuality/sexualities in the breastfeeding context, which is justified by the fact that it is a period marked by dedication and accumulation of activities. Caring for their child(ren), breastfeeding, domestic issues, and even professional work are some of these accumulations, where nursing mothers do not always have a support network.

Faced with this various transformations associated with physical, hormonal, structural, emotional, and social issues, nursing mothers build representations of sexuality/sexualities during the breastfeeding period, judging it/them as difficult. These representations were elaborated with greater strength by the multiparous women and by those with up to six months as nursing mothers, in other words, women in the initial phase of breastfeeding and the postpartum period, which brings about the justification function of this representation. It is through this function that it is possible to convey an image of a given group to a phenomenon in the interaction context, allowing to explain and justify stances, behaviors and practices^(23)^.

Faced with the negative evaluative dimension of the representation of sexuality/sexualities in breastfeeding, the evocations of care measures and respect emerged, as a necessity for this phenomenon at this moment in life, which requires new adaptations and a support network, especially at breastfeeding initiation and for younger women, as it was strongly represented by the youngest participants and by those with six months as nursing mothers.

A search carried out with nursing mothers of Iran and Switzerland corroborates the importance of the support network, especially the couple interaction, so that they can experience sexuality in the breastfeeding context in a positive way^(24)^. In another study, carried out in Spain, it is pointed out that this interaction can be stimulated by the partners’ participation in sexual and reproductive health consultations, which should aim at contextualizing the practice of sexuality, as a proposal to improve the quality of intimate relationships, as a psychosocial benefit experienced by nursing mothers^(25)^.

Nothing is static in the scope of culture and of representations; in this sense, the results showed certain movement marked by normalization and naturalization of sexuality and/ sexualities during the breastfeeding process. “Normal” was the evocation with the greatest contribution to explaining sexuality/sexualities during this period among the nursing mothers with more than one year as such. This interpretation can be supported on the body returning to the conditions prior to pregnancy and on the nursing mothers’ adaptations to the new requirements and changes in their routines, maybe because they have already spent a longer period as a nursing mother.

The roles of mother, wife, woman, nursing mother, and professional seem to have adjusted, or to be in such process, after one year of the breastfeeding experience, which makes them deem sexual relations as normal. With this, they show the conventional and prescriptive nature of the SRs that model objects, people or events, according to language, time and culture of a given social group^(9)^.

## CONCLUSION

The analysis of the most relevant evocations that emerged from the participants’ responses, based on the correlations between the inducing stimuli and the fixed variables, allowed us to verify the existence of a diversity of meanings attributed to sexuality/sexualities in the breastfeeding period, in the different contexts of a woman’s life, whether related to the time she has been nursing, her age group, and even the parity classification. This diversity makes us include this term in the plural, asserting the multiplicity of representations in relation to this phenomenon.

Sexual intercourse was the most significant expression of sexuality/sexualities, which was consequently reflected in the elaboration of this phenomenon in the breastfeeding context, anchored in cultural gender-based relationships. These constructions are explained from the difficulties experienced during this period, which are naturalized or end up exerting a negative experience on these women’s sexual and reproductive health over time.

The study limitation corresponds to the fact that it only investigates a single group of nursing mothers from an HMB in northern Brazil. Broader studies that include diversified realities are desirable, to verify the different representations of the object under study.

The understanding of sexuality/sexualities in the breastfeeding context as an SR phenomenon can support Nursing and Health Care models, aligned and centered on nursing mothers’ experiences and social thoughts, as well as recommending efforts to improve the sexual and reproductive health care network for this population segment.
